# Some Points on Alcoholism

**Published:** 1909-03-27

**Authors:** 


### Resident Medical Officers' Department.

### SOME POINTS ON ALCOHOLISM.

By A Hospital Resident.

One of the most important aspects of the question of the excessive use of alcohol is the case of the individual who does not know that he is taking more than is good for him. The case is important, first because of the insidious nature of the disorder, and secondly because it is a condition which is but little recognised, seldom thought about, and frequently shunned by anyone who comes in contact with it. Yet there are many lives lost annually from cirrhosis of the liver, or from alcoholic myocardial degeneration in people who have never known that they are taking an excessive amount of alcohol, who have certainly never been actually drunk, and who very likely have never even been "fuddled.'

Such people will tell you straight out that they have been in the habit of taking four, six, or even eight pints of beer a day, and from their very manner it is possible to tell at once that they are neither in any way ashamed of the fact, nor aware of the harm it may have done them, yet they may at that moment have well-marked evidence of cirrhosis of the liver, have but lately recovered from a serious attack of hæmatemesis, or be in a very critical condition from failure of the heart.

The condition is one of extreme importance to the members of the medical profession, whether they are general practitioners or specialists, or resident medical officers. Chronic alcoholism is a factor which must be carefully estimated in advising a patient to undergo an operation, or in giving a prognosis in cases of acute illness. The only remedy appears to be the gradual education of the layman by the profession as to the danger that he is incurring. It is not a phase of the alcohol problem that can in any way be touched by legislation; and yet the individual cannot help himself, for he does not know that there is anything wrong. Horsley and Sturge in their book on alcohol have shown the effects on mental and physical work of taking alcohol in so-called dietetic quantities. The case we are discussing now is not that of the man whose ideas of dietetic quantities are broad, or who may be assumed to be taking alcohol in quantities just in excess of dietetic principles; it is the intermediate state between this last and the chronic tippler.

Horsley and Sturge showed that the habitual use of alcohol even in small quantities prevents the individual from attaining the same high pitch of mental activity or physical endurance as the teetotaller can. Such an amount, while depressing the higher faculties and so impairing the wage-earning power of the individual, has little or no deleterious action on his physical existence. In somewhat increased quantities alcohol seems to have the further effect of insidiously damaging vital tissues in the ways mentioned above. In the next stage the mental impairment has become obvious to the impressions of the observer without the necessity of carefully planned experiments; this is the condition of the chronic tippler. It is the intermediate of these three stages that we would lay stress upon, as being little recognised, and so difficult to cope with.

Let us now consider how this condition is to be treated. The first point is to recognise clinically this variety of case, the second to realise in what class such cases are particularly liable to occur. The class is that of men who have to work in hot atmospheres, and who therefore take a large amount of fluid to keep themselves cool; among such men the idea is very prevalent that to drink water in large quantities is dangerous to the health. Their inclination bids them follow what appears to them their good, and they turn to beer. No doubt among large works and factories the provision of water with meal in it has done much good, yet there remains a large number of individuals still ignorant of the use of this mixture, and among them this form of alcoholism is not infrequently seen. For the rest the amount of alcohol which it has been the fashion to look upon as within limits has decreased considerably in the past century, and will probably continue to decrease; with this decrease this class of case will tend to die out.

Turning from the general to the individual, men and women are frequently seen in whom one suspects this condition; they suffer from chronic indigestion, the skin has a greasy, shiny appearance, and they seem to be covered with a flabby connective tissue in excess of what they should. A habit of rapidly protruding the tongue as though to moisten the lips, frequently repeated, is most suggestive.

The treatment in cases such as these is without doubt to ask them about the amount of alcohol they take, to tell them it is too much for them, and to advise them to be teetotal. Yet the difficulties are very great. It is hard sometimes not to look upon them as drunkards, it is harder still to prevent the patient from thinking the doctor looks upon him as a drunkard. The difficulty is two-fold: there is the natural hesitancy a man has in hurting another, and the feeling that the patient will suffer mental pain and loss of self-esteem in being told he takes too much alcohol; and there is the second thought, that very likely the patient will not believe the statement, will be very angry, and refuse all further advice. In this way there is a tendency to shirk the subject, yet, no doubt, it should be faced when one comes across it, however difficult it may seem. It is a situation needing much tact, but it should come easier if it is fully realised that frequently these patients do not know they are taking too much alcohol, and that they are quite willing to be open on the subject if they are approached on it.

